# AHNAK2 is a biomarker and a potential therapeutic target of adenocarcinomas: AHNAK2 is a biomarker for adenocarcinomas

**DOI:** 10.3724/abbs.2022112

**Published:** 2022-08-19

**Authors:** Meng Xu, Anyi Cheng, Liya Yu, Wei Wei, Jinpeng Li, Cheguo Cai

**Affiliations:** 1 Department of Thyroid and Breast Surgery Zhongnan Hospital of Wuhan University; Medical Research Institute Frontier Science Center for Immunology and Metabolism Wuhan University Wuhan 430071 China; 2 Department of Thyroid and Breast Surgery Zhongnan Hospital of Wuhan University Wuhan 430071 China

**Keywords:** WGCNA, adenocarcinoma, squamous carcinoma, AHNAK2

## Abstract

Adenocarcinoma is the second largest histological type of cervical cancer, second only to cervical squamous cell carcinoma. At present, despite the clinical treatment strategies of cervical adenocarcinoma and cervical squamous cell carcinoma being similar, the outcome and prognosis of cervical adenocarcinoma are significantly poor. Therefore, it is urgent to find specific biomarker and therapeutic target for cervical adenocarcinoma. In this study, we aim to reveal and verify the potential biomarkers and therapeutic targets of cervical adenocarcinoma. Weighted correlation network analysis (WGCNA) reveals the differentially-expressed genes significantly related to the histological characteristics of the two cervical cancer subtypes. We select the genes with the top 20 significance for further investigation. Through microarray and immunohistochemical (IHC) analyses of a variety of tumor tissues, we find that among these 20 genes, *AHNAK2* is highly expressed not only in cervical adenocarcinoma, but also in multiple of adenocarcinoma tissues, including esophagus, breast and colon, while not in normal gland tissues. *In vitro*, *AHNAK2* knockdown significantly inhibits cell proliferation and migration of adenocarcinoma cell lines. *In vivo*, *AHNAK2* knockdown significantly inhibits tumor progression and metastasis of various adenocarcinomas. RNA-sequencing and bioinformatics analyses suggest that the inhibitory effect of *AHNAK2* knockdown on tumor progression is achieved by regulating DNA replication and upregulating Bim expression. Together, we demonstrate that AHNAK2 is a biomarker and a potential therapeutic target for adenocarcinomas.

## Introduction

Epithelial solid tumors are usually divided into squamous cell carcinomas (SCCs) and adenocarcinomas (ACs) according to pathological criteria such as origin, morphological structure, and special biomarkers. SCCs derived from squamous epithelium have common histological characteristics, and they show squamous differentiation through the formation of keratin pearls [1]. ACSs occur in glandular tissue and are the largest group of human epithelial malignancies [2]. Although ACs and SCCs from the same tissue present obvious molecular heterogeneity [ 3, 4] , SCCs from different positions (tissues), such as cervix, lung, esophagus, and others, share many common gene mutations, and the mutation rate is significantly higher than that of adenocarcinomas from the same tissue [ 5– 7] , indicating that tumors from different tissues of the same histological type may have similar mutation landscape, and tumors from different histological types of the same tissue may have different mutations. Therefore, it is of great interest to study diagnosis biomarkers and therapeutic targets according to tumor histological types.

Cervical cancer is one of the cancers with the highest incidence rate and mortality in women [8]. The most common histological subtypes of cervical cancer are squamous cell carcinoma and adenocarcinoma, accounting for about 70% and 25% of cervical cancer patients, respectively [9] . In the past decade, early screening and vaccination programs have effectively reduced the overall incidence of cervical cancer. However, recent epidemiological studies indicated that the incidence of cervical adenocarcinoma is increasing [10]. Compared with cervical squamous cell carcinoma, the incidence of cervical adenocarcinoma is low and is not always associated with high-risk subtypes of human papillomavirus (HPV) infection [ 11, 12] . In addition, compared with that of squamous cell carcinoma, the survival of adenocarcinoma patients was significantly shorter [13]. Of note, the early screening of cervical cancer through HPV detection combine with or without cytology survey is effective for the prevention and treatment of cervical squamous cell carcinoma, but the screening program for cervical adenocarcinoma is not always as effective as for squamous carcinoma [10]. However, despite many differences between the two cervical cancer subtypes, according to the guidelines of the international federation of gynecology and obstetrics (FIGO) and the national comprehensive cancer network (NCCN), patients with cervical squamous cell carcinoma and cervical adenocarcinoma receive almost the same treatment strategies, including surgery, adjuvant radiotherapy or chemoradiotherapy [14]. This obviously does not meet the requirements of personal and precise treatment at present. Therefore, the investigation of the differences in gene expression patterns and pathogenesis between squamous cell carcinoma and adenocarcinoma will provide a basis for the discovery of novel diagnostic markers and therapeutic targets, and provide guidance for the precise treatment of cervical cancer.

Biomarker genes can be obtained by differential gene analysis and screening of data from public databases or samples sequencing data [ 15, 16] . However, considering that the occurrence and progression of tumor is the result of the collaborative work of functional gene sets [17], it is more purposeful and effective to systematically analyze and screen gene sets related to a certain feature of the tumor. Weighted Correlation Network Analysis (WGCNA) is a system biology approach based on the correlation of gene expression, which can be used to establish highly collaborative gene modules [18]. Using this approach, a pair-wise Pearson correlation matrix is created for all genes. β power is selected based on the criterion of “scale-free network topology”, and is further used to establish the adjacency matrix (Network). Using the established co-expression network, the “modules” composed of genes with similar expression and connection patterns are detected. These modules are then correlated with biological covariates to find genes associated with tumor phenotype. This method has been applied to the identification of functional modules of co-expressed genes at various developmental stages of human and mouse embryos [19], the discovery of molecular characteristics of stem cells in the neurogenic area of adult mouse forebrain [20], and the screening of key genes related to telomerase in cancers [21].

In this study, we aim to reveal diagnosis biomarkers and therapeutic targets of adenocarcinoma by using omics and bioinformatics analyses. We analyzed The Cancer Genome Atlas (TCGA) database, identified specific genes of cervical adenocarcinoma by WGCNA, and further investigated the function of these genes in cervical adenocarcinoma and breast cancer tumor models. We described AHNAK2 as a biomarker and therapeutic target for adenocarcinomas.

## Materials and Methods

### Bioinformatic analysis

The RNA sequencing level 3 data of malignancies (cervical carcinoma, breast carcinoma, colorectal carcinoma, lung adenocarcinoma, and pancreatic cancer) and the matched clinical information of patients were retrieved from the TCGA database. Identification of the hub genes related to histological type was conducted with the ‘WGCNA’ R package. Kaplan-Meier curve analysis was realized by the R ‘survival’ package, the optimal cut-off value of high-risk and low-risk calculated by X-tile [22]. Differential gene expression (DEG) analysis was performed with the ‘Limma’ R package, setting a log _2_ |Fold Change| ≥1.5, *P*<0.05, and a false discovery rate (FDR)<0.05 as the cutoff values. The visualization of DEGs was completed with the ‘Complex Heatmap’ R package. Kyoto encyclopedia of genes and genomes (KEGG) pathway enrichment analysis of the DEGs were constructed using Gene Set Enrichment Analysis (GSEA) software (version 4.1.0). The R package used in this study is version 4.0.5.

### Patient samples

Cervical carcinoma tissue-microarray (#HUteS168Su01, 168 cases/168 points) including 4 cervical adenocarcinoma tissues purchased from Shanghai OutDo Biotechnology Company ( http://www.superchip.com.cn/biology/tissue.html) (Shanghai, China). Breast carcinoma tissue-microarray (#BR804b, 40 cases/80 points) containing cancer and para-cancerous tissues, colorectal carcinoma tissue-microarray (# C01801, 90 cases/180 points) including cancer and para-cancerous tissues, cervical adenocarcinoma tissue-microarray (#CR246, 20 cases/44 points) containing cancer and para-cancerous tissues were purchased from Biomax, Inc. ( https://www.biomax.us/) (Derwood, USA). In addition, 26 archived formalin fixed tissues of cervical adenocarcinoma tissues were collected from Zhongnan Hospital of Wuhan University (Wuhan, China) and Longgang District Maternal & Child Healthcare Hospital of Shenzhen (Shenzhen, China), and tissue-microarray was made in Shanghai OutDo Biotechnology Company. The study was approved by the Medical Ethics Committee of Zhongnan Hospital of Wuhan University (CHiCTR180001247), and all patients signed informed consent forms. The imperfect tissues on the microarray were rejected during the analysis.

### IHC staining and IHC quantifications

Formalin-fixed paraffin-embedded tissue-microarrays were incubated with anti-AHNK2 antibody (#HPA002940; 1:300 dilution; Sigma, St Louis, USA) overnight at 4°C. Antibody binding was detected by incubation with an anti-rabbit biotinylated secondary antibody for 30 min at room temperature. DAB (3,3’-diaminobenzidine) color reaction was performed according to the procedure (#K500711-2; Agilent, Santa Clara, USA). In addition, some of the immunohistochemical results were downloaded from the Human Protein Altas ( https://www.proteinatlas.org/). The immunostaining was assessed and scored by two independent examiners. The score was assessed according to the intensity of the staining as 0+ (negative), 1+(low), 2+(medium) and 3+(high), and the extension of the staining as 0+(negative), 1+(10%–25%), 2+(25%–50%), 3+(50%–75%) and 4+(75%–100%). The final score was calculated by multiplying “intensity score”×“staining area score”. Take score=4 as the cutoff value to divide the samples into AHNAK2-high and AHNAK2-low groups.

### Cell culture and transfection

Cervical carcinoma cell lines Hela, breast carcinoma cell lines MDA-MB-231, and HEK293T were purchased from American Type Culture Collection (ATCC; Manassas, USA). Cells were cultured in Dulbecco’s modified Eagle’s medium (DMEM; Invitrogen, Carlsbad, USA) or RPMI-1640, supplemented with 10% FBS (Gibco, Carlsbad, USA), 100 units/mL penicillin, and 100 μg/mL streptomycin (Gibco). Lentiviral vector pLKO.1 (Addgene, Watertown, USA) whose backbone with GFP was allowed the ectopic expression of scramble shRNA (5′-TCCTAAGGTTAAGTCGCCCTCG-3′; Sangon Biotech, Shanghai, China) or AHNAK2 shRNA (Sh#1- *h*- *AHNAK2*-F 5′-CCGGGGTGCGAGTACACGATTTAAACTCGAGTTTAAATCG TGTACTCGCACCTTTTTG-3′, Sh#1- *h*- *AHNAK2*-R 5′-AATTCAAAAAGGTGCGAGTACACGATTTAAACTCGAGTTTAAATCGTGTACT CGCCC-3′; and Sh#2- *h*- *AHNAK2*-F 5′-CCGGACGCACAGAGGAAGGATTAAACTCGAGTTTAATCCTTCCTCTGTGCGTTTTTTG-3′, Sh#2- *h*- *AHNAK2*-R 5′-AATTCAAAAAACGCACAGAGGAAGGATTAAACTCGAGTTTAATCCTTCCTCTGTGCGT-3′; Sangon Biotech), vesicular stomatitis virus G (Addgene), and packaging plasmid Delta 8.9 (Addgene) were co-transfected into HEK293T cells through conventional calcium phosphate method to produce lentivirus. GFP ^+^ cells were selected with BD Accuri C6 Flow cytometer (Franklin Lakes, USA).

### Real-time qPCR

Total RNA was isolated from cells using Trizol (Invitrogen) according to the manufacturer’s instructions, and the PrimeScript RT Master Mix kit (TaKaRa, Dalian, China) was used for the reverse transcription reaction. The quantitative polymerase chain reaction (RT-pPCR) assay was conducted according to the instructions for the Mon Amp ^TM^ SYBR ^®^ Green qPCR Mix kit (Monad, Suzhou, China) on the Bio-Rad CFX system (Hercules, USA) with the following conditions: pre-denaturation at 95°C for 30 s; denaturation at 95°C for 5 s, annealing at 60°C for 10 s and extension at 72°C for 30 s, and repeated denaturation, annealing and extension for 40 cycles. The relative expression of different genes was calculated using the 2 ^−△△CT^ method. *GAPDH* served as the internal reference. qPCR primers used in this study are shown in Table 1.

**Table:** **Table 1** Sequences of primers used in this study

Gene	Primer sequence (5′→3′)
*h-AHNAK2*	Forward GAGAAGGAGGACACGGATGTTGC
	Reverse CCCCGCTTGCTCTTTATGGATTG
*h-PCNA*	Forward TGGAGAACTTGGAAATGGAAA
	Reverse GAACTGGTTCATTCATCTCTATGG
*h-RFC1*	Forward TTGTCATGGGTCGTGATAGTGG
	Reverse CCTGGCATAGTCCGAATCAGAT
*h-RFC3*	Forward CTTCCTTCACAACTGGCTCAT
	Reverse TGCAGGCTTCACACATAAGCA
*h-MCM4*	Forward TGAACCTCTATACATGCAACGAC
	Reverse CAGGGTAACGGTCAAAGAAGATT
*h-MCM6*	Forward GAGGAACTGATTCGTCCTGAGA
	Reverse CAAGGCCCGACACAGGTAAG
*h-POLA1*	Forward AAAGATCCATTGGAGCTTCACC
	Reverse TCAGCACGTTTAAGAGGAACAG
*h-POLD3*	Forward GAGTTCGTCACGGACCAAAAC
	Reverse GCCAGACACCAAGTAGGTAAC
*h-POLE2*	Forward TGAGAAGCAACCCTTGTCATC
	Reverse TCATCAACAGACTGACTGCATTC
*h-DNA2*	Forward AGAGCTGTCCTGAGTGAAACT
	Reverse GAAACACCTCATGGAGAACCG
*h-RNASEH2B*	Forward TAACCCCTGTTCAGGAGAAGG
	Reverse ACACGTTATCCACCACAACTTG
*h-PRIM2*	Forward AATGCTTCCTACCCTCATTGC
	Reverse AGCTCACTCTCCAACTTACTCTG
*h-GAPDH*	Forward GGAGCGAGATCCCTCCAAAAT
	Reverse GGCTGTTGTCATACTTCTCATGG

### Immunofluorescence staining

Cells were plated in 24-well dishes and cultured for 24 h. At the endpoint, cells were fixed in 4% ice-cold paraformaldehyde (PFA) for 30 min and permeabilized with 0.5% Triton-X-100 for 30 min at room temperature. After being blocked with 5% BSA in PBS, fixed cells were incubated with the anti-AHNAK2 antibody (1:300 dilution; Sigma) at 4°C overnight, followed by incubation with Cy3-conjugated anti-rabbit IgG secondary antibody (1:500; Jackson ImmunoResearch, West Grove, USA). A Zeiss microscope (Oberkochen, Germany) was used for imaging.

### Cell proliferation assay

Cells were seeded into a 6-well plate and assessed by counting the number of cells every 48 h and growth curves were plotted. For colony formation assay, cells were seeded in a six-well plate at a density of 1000 cells/well and then cultured for about 2 weeks. The colonies were then fixed with 4% paraformaldehyde and stained with 0.1% crystal violet for 30 min. Stained cell colonies were washed with phosphate-buffered saline (PBS) three times and dried. Images were obtained with a digital camera (Sony, Tokyo, Japan) and the number colonies containing more than 50 cells were counted.

### Cell migration and invasion assays

The cell migration ability was detected by wounding healing assay. Cells were seeded in a 6-well plate and allowed to form a cell monolayer after 24 h of culture with the fresh complete medium. Then a vertical scratch was made with a P200 pipette tip and the medium was replaced by medium without FBS. Cell migration in the wound area was monitored under a microscope and images were captured and analyzed with ImageJ software. The invasion ability of cells was detected using the trans-well chambers (8 μm pore size; Corning, New York, USA) pre-coated with Matrigel (#354234; Corning) in a 24 well plate. Cells suspended in culture medium without FBS were seeded into the upper chambers, whereas the bottom chamber was filled with a fresh complete medium containing 10% FBS. After 24 h of incubation, invaded cells were fixed with 4% paraformaldehyde (PFA) and stained with 0.1% crystal violet. Three visual fields were randomly selected and the numbers of invaded cells were counted under a microscope (Zeiss).

### Animal experiments

The animal experiments were approved by the Animal Care and Use Committee of Wuhan University. Mice were kept in a standard animal facility with free access to food and water. Tumorigenicity *in vivo* was performed by subcutaneous transplantation and orthotopic transplantation. Cell suspensions were injected subcutaneously (100 μL of 10 ^6^ cells, PBS:Matrigel=2:1) into the posterior flanks or orthotopically (10 μL of 10 ^6^ cells, 50% FBS/PBS:Matrigel=1:1) in the fourth mammary fat pad of four to six-week-old immunodeficient nude female mice (Charles River, Beijing, China). Tumor volume was monitored three times per week using a caliper and calculated by the following formula: tumor volume= length×width ^2^×0.52. Mice were sacrificed when tumors reached a volume of approximately 0.2 cm ^3^. Cancer cells (10 ^5^ cells), which were stably transduced with luciferase before injection to allow for bioluminescent imaging (BLI) *in vivo*, were suspended in 100 μL PBS and injected into the left cardiac ventricle of six-week-old immunodeficient nude female mice or female Balb/c mice. Animals were monitored and imaged weekly by Spectral Instrument Imaging (The Cold Spring Harbor Laboratory, New York, USA) *ex vivo* after 15 min of intraperitoneal injection of luciferase (#40902ES01; Yepsen, Shanghai, China). The BLI value was detected by Living Image® Software 4.4 (Perkin Elmer, Waltham, USA). For intravenous injections of the tumor cells, 10 ^6^ cells containing GFP fluorescence were suspended in 100 μL PBS and injected into the lateral tail vein of nude mice, and then the mice were sacrificed when the weight of the mice was obviously decreased and in a poor state. The metastatic lesions in the lung of mice were photographed and counted under a stereoscope (Leica, Wetzlar, Germany).

### RNA-sequencing analysis

Total RNA was extracted from AHNAK2-deficient tumor cell lines and the control cells using the Trizol reagent. After the total RNA samples were qualified by nanodrop (Thermo Fisher, Waltham, USA), 5 μg of total RNA was taken for subsequent libraries construction. In brief, oligo (DT)-loaded beads were used to enrich mRNA, and then fragmentation buffer was added to break the obtained mRNA into short fragments. The first strand of cDNA was synthesized with six base random primers, and then the second-strand synthesis was performed by adding buffer, dNTPs, and DNA polymerase I. Agarose gel electrophoresis and Qubit 2.0 were used to test and quantify the constructed library and determine the concentration of the library. Suitable samples were used for sequencing with a HiSeq2000 sequencer (Illumina, San Diego, USA). The raw data of sequencing were filtered to obtain high-quality sequencing data (clean data). The clean data were compared with the reference genome of the project species to obtain comprehensive transcriptional information. The RNA sequencing and library construction were completed by the Seqhealth Company (Wuhan, China). The RNA-sequencing data have been deposited in NCBI’s Gene Expression Omnibus and are accessible through GEO Series accession number GSE179982.

### EdU staining

Cells were seeded into a 24-well plate. After 24 h of culture, cells were exposed to 10 μM of 5-ehtynyl-2′-deoxyuridine (EdU; Shanghai Donghuan Biotech, Shanghai, China) for 2 h at 37°C, and then the cells were fixed in 4% PFA. After permeabilization with 0.5% Triton-X, the cells were reacted with 1× EdU staining cocktail for 30 min. Then, cells were stained with 1× Hoechst 33342 for 30 min and examined under a fluorescence microscope (Zeiss).

### Cell apoptosis assay

The cell apoptosis was evaluated using the Annexin V Alexa Fluor 647/PI Apoptosis Detection kit (Solarbio, Beijing, China) according to the manufacturer’s instructions. Cells were seeded into a 6-well plate. After treatment, all the cells were collected, briefly washed twice with PBS, and resuspended in binding buffer. Then, Annexin V Alexa Fluor 647 and PI were added to the cell suspension and incubate at room temperature for 15 min in the dark. Finally, cells were analyzed by flow cytometry on the BD LSRFortessaX20 flow cytometer (BD) within 1 h.

### Western blot analysis

Proteins were extracted from cells using ice-cold RIPA lysis buffer containing 1 μM phenylmethanesulfonyl fluoride (PMSF; Sigma). Equal amounts of protein were separated by SDS-PAGE and blotted onto PVDF membranes. After blocking, the membranes were probed with an anti-Bim mAb (#2933S; 1:1000 dilution; Cell Signaling Technology, Beverly, USA) overnight at 4°C. A GAPDH mAb (#5174S; 1:1000 dilution; Cell Signaling Technology) was used as the control. Then the PVDF membranes incubated with HRP-conjugated secondary antibody (1:10000 dilution; Cell Signaling Technology) for 1 h at room temperature. The protein bands were visualized using the Enhanced chemiluminescent (ECL) detection reagents (Monad).

### Statistical analysis

All experiments were repeated three times unless otherwise specified. Statistical analysis was performed with GraphPad Prism 7.0 software. The comparisons between groups were performed using unpaired Student’s *t*-test. Data were presented as the mean ± standard deviation (SD). *P*<0.05 was considered statistically significant.

## Results

### The top 20 genes associated with histological types of cervical cancer

**Figure 1:**
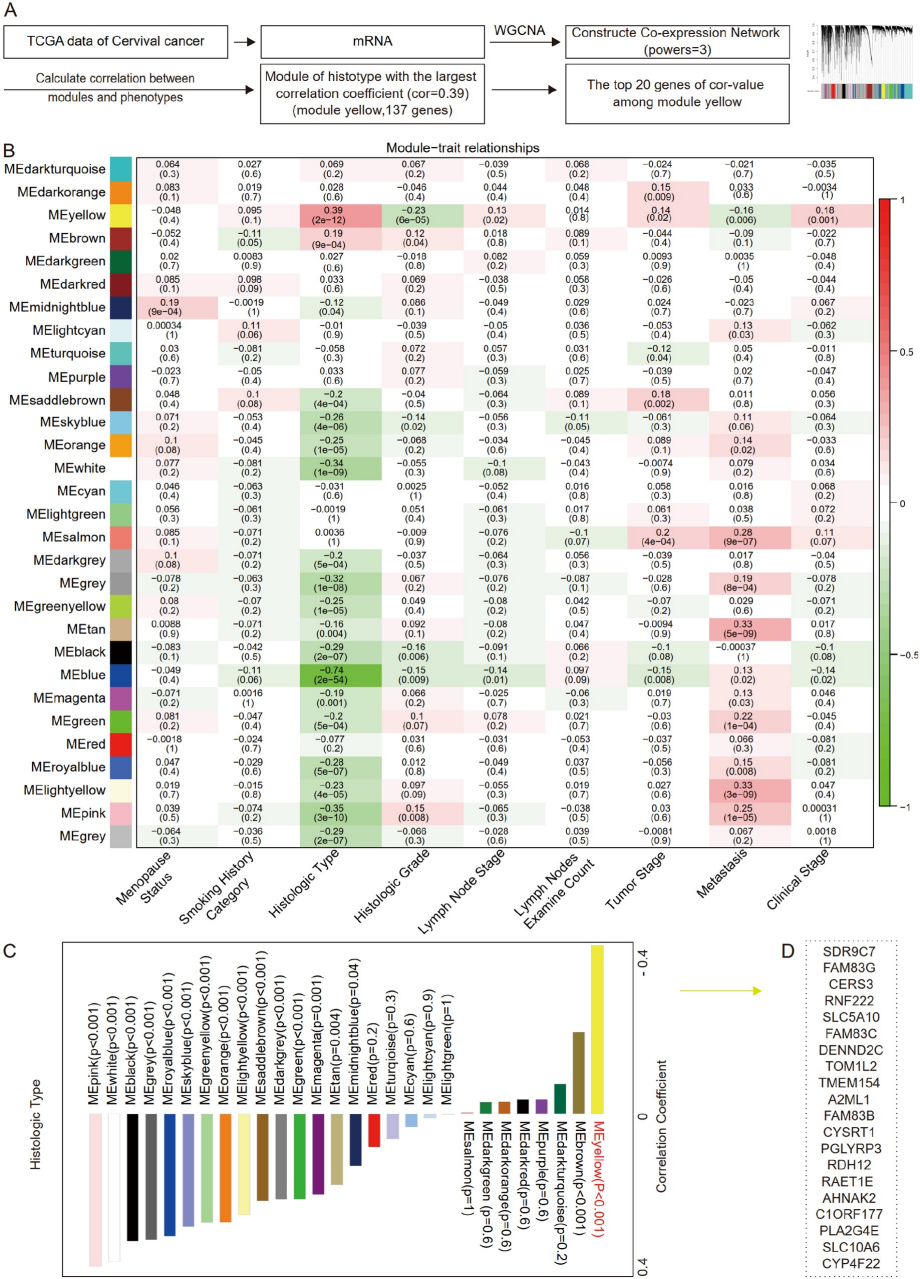
Figure 1 The top 20 genes associated with the histological types of cervical cancer (A) Overall workflow of the screening of target genes (WGCNA: weighted gene co-expression network analysis; Cor-value: correlation coefficient value). (B) Heatmap of the relationship between module eigengenes and selected clinical traits of cervical cancer. (C) Correlation coefficients and P-values between the module eigengenes (ME) and histologic type. (D) The top 20 genes in the ME yellow.

To identify the key differential genes related to histologic subtypes in cervical cancer, we performed data-mining analyses from TCGA datasets ( http://www.cbioportal.org/datasets), including mRNA sequencing data and clinical outcome data (20,440 genes, 306 samples) ( Figure 1A). The correlation coefficient between any two genes was calculated, and the top 25% genes with the largest value were selected as the input data set of WGCNA (4585 genes, 306 samples). Taking 3 as the soft threshold power to construct the co-expression matrix, 4585 genes were divided into 30 modules represented by different colors through dynamic tree-cutting algorithm. To make the screening scope of key genes more concentrated, all the major clinical characteristics of cervical cancer patients (menopause status, smoking history category, histologic type, histologic grade, lymph node stage, tumor stage, metastasis, and clinical stage) were selected to calculate the Pearson correlation coefficient with different modules, and the module mostly relevant to histological type was obtained (yellow module, Cor=0.39; *P*<0.001; Figure 1B,C). The yellow module contained 137 genes, among which the top 20 genes of the highest correlation coefficient value were *SDR9C7*, *FAM83G*, *CERS3*, *RNF222*, *SLC5A10*, *FAM83C*, *DENND2C*, *TOM1L2*, *TMEM154*, *A2ML1*, *FAM83B*, *CYSRT1*, *PGLYRP3*, *RDH12*, *RAET1E*, *AHNAK2*, *C1ORF177*, *PLA2G4E*, *SLC10A6*, and *CYP4F22* ( Figure 1D and Supplementary Table S1). Therefore, the top 20 genes significantly associated with histological types of cervical cancer were obtained.

### AHNAK2 is a biomarker of cervical adenocarcinoma

**Figure 2:**
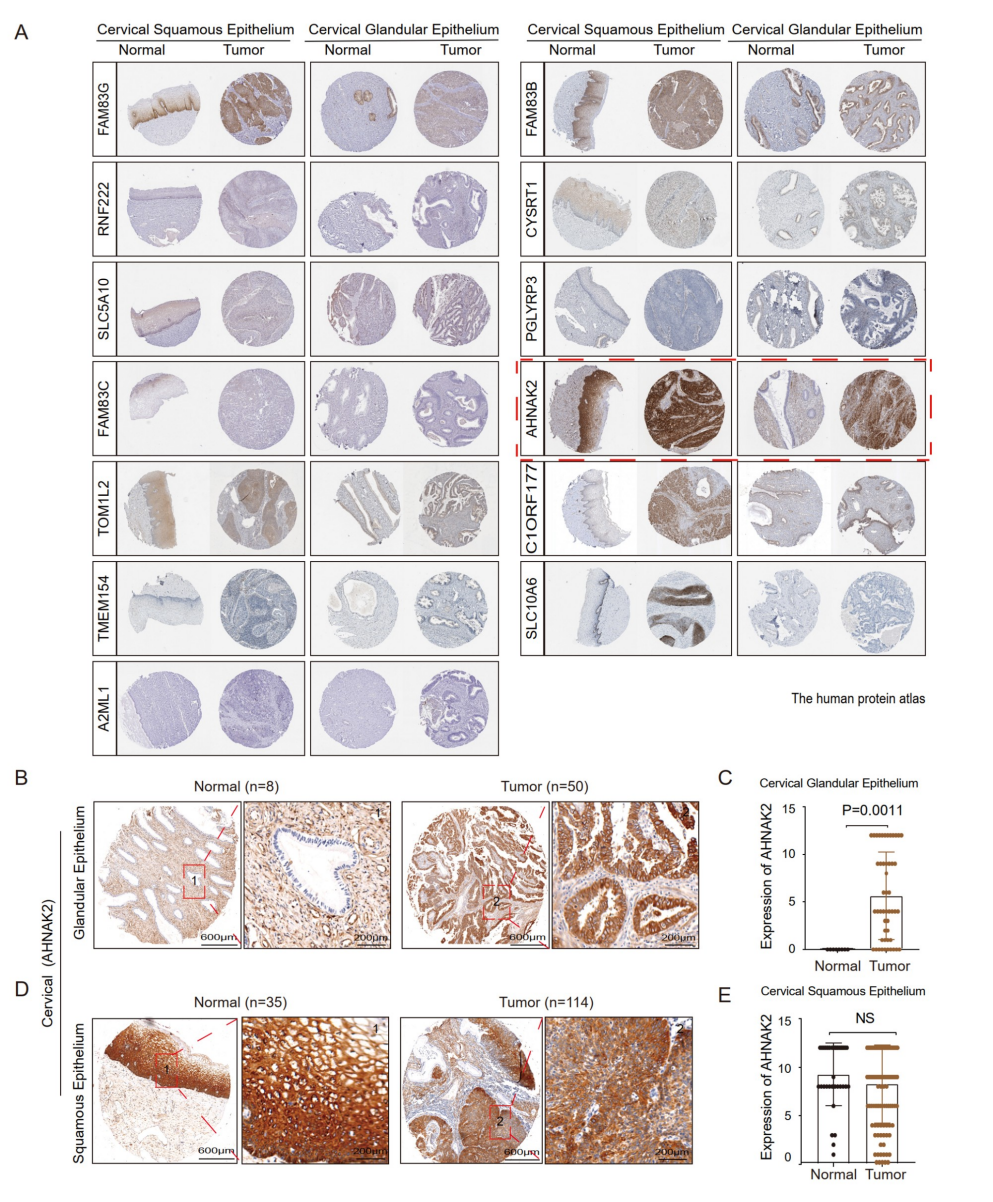
Figure 2 AHNAK2 is highly expressed in cervical adenocarcinoma tumors (A) Representative IHC results of the top genes in cervical squamous tumor and adenocarcinoma and corresponding normal tissues from Human Protein Atlas (FAM83G, RNF222, SLC5A10, FAM83C, TOM1L2, TMEM154, A2ML1, FAM83B, CYSRT1, PGLYRP3, AHNAK2, C1ORF177, and SLC10A6). (B,C) Representative IHC staining images of AHNAK2 in a tissue microarray of the cervical glandular epithelium ( n=8) and adenocarcinoma ( n=50). (D,E). Representative immunohistochemistry staining images of AHNAK2 in the cervical squamous epithelium ( n=35) and squamous cancer ( n=114).

The Human Protein Atlas (HPA, https://www.proteinatlas.org/) is a Swedish-based program launched in 2003 to map all the human proteins in cells, tissues, and organs using an integration of various omics techniques. To verify the expression pattern of those 20 genes revealed from WGCNA of cervical cancer, we further compared the expressions of the 20 genes in carcinoma and non-carcinoma tissues of cervical cancer using immunohistochemistry (IHC) data from HPA. In the IHC data of HPA, there is a lack of carcinoma or corresponding normal tissues of several candidate genes including *SDR9C7*, *RDH12*, *RAET1E*, *CERS3*, *PLA2G4E*, *CYP4F22*, and *DENND2C*. The expressions of the remaining 13 genes in cervical cancer were shown in Figure 2A. We observed that there was no significant difference in the expressions of *FAM83G*, *RNF222*, *SLC5A10*, *FAM83C*, *TOM1L2*, *TMEM154*, *A2ML1*, *FAM83B*, *CYSRT1*, *PGLYRP3*, *C1ORF177* and *SLC10A6* in cervical squamous cell carcinoma and cervical adenocarcinoma compared with those in the normal squamous epithelium and normal glandular epithelium, respectively ( Supplementary Figure S1). Interestingly, AHNAK2 displayed a unique expression pattern. Its expression in cervical adenocarcinoma was significantly higher than that in the normal glandular epithelium, but there was no significant difference between cervical squamous cell carcinoma and normal squamous epithelium, suggesting that AHNAK2 may be a potential biomarker of cervical adenocarcinoma.

To verify the expression pattern of *AHNAK2* shown in the HPA database, we performed immunohistochemical staining (IHC) analysis using a tissue microarray containing cervical squamous cell carcinoma, adenocarcinoma, and normal tissues. Consistent with the HPA database, IHC analysis showed that the expression of AHNAK2 was significantly higher in cervical adenocarcinoma ( *n*=50) than in the normal glandular epithelium ( *n*=8) ( *P*<0.01; Figure 2B,C and Supplementary Figure S2A). However, AHNAK2 was highly expressed in the normal cervical squamous epithelium ( *n*=35) and cervical squamous cell carcinoma without significant difference ( *n*=114) ( *P*-value: NS; Figure 2D,E and Supplementary Figure S2B). Together, these data demonstrated that AHNAK2 is a potential biomarker of cervical adenocarcinoma.

### AHNAK2 is a biomarker for various adenocarcinomas

**Figure 3:**
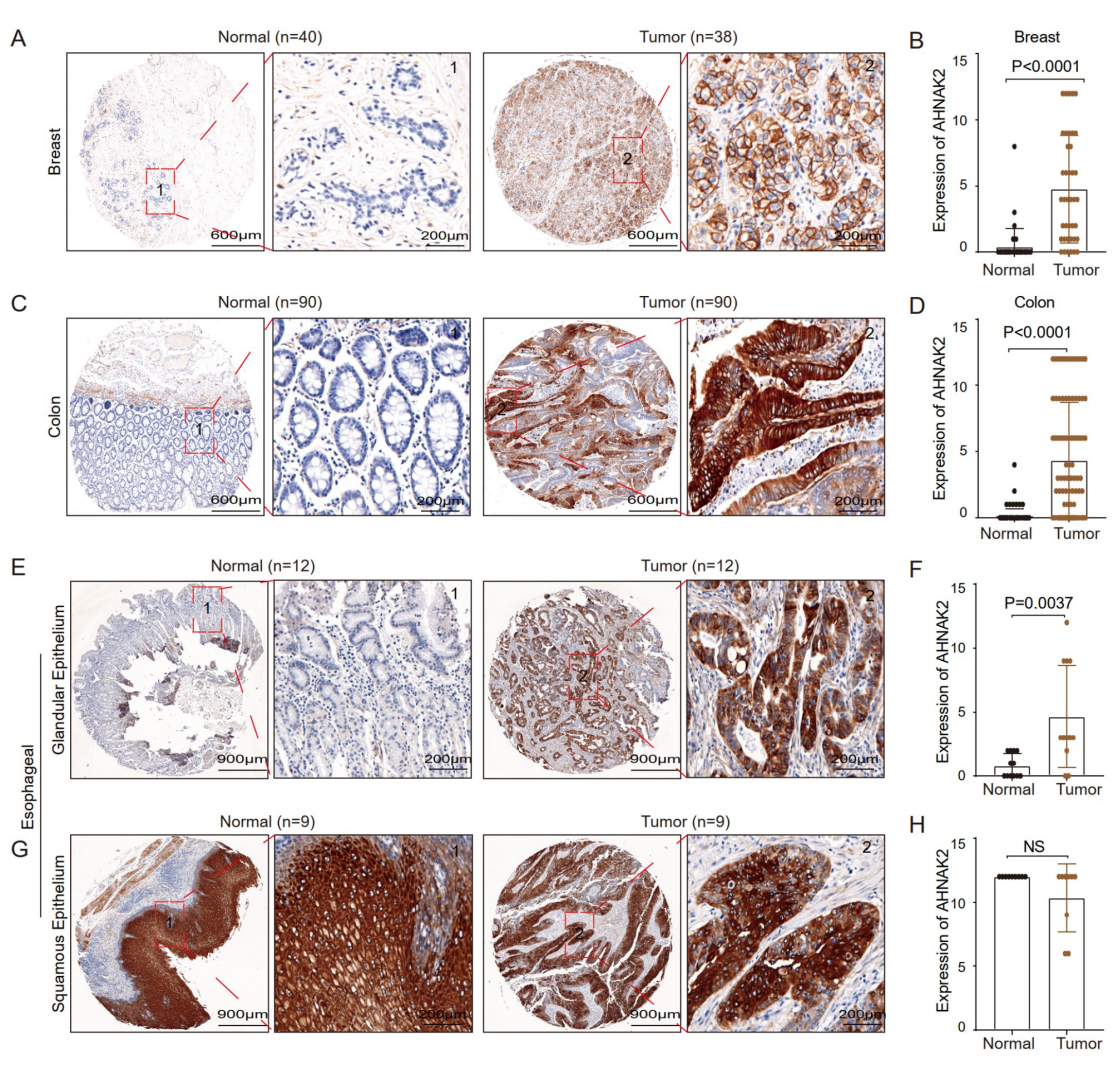
Figure 3 AHNAK2 is highly expressed in various adenocarcinomas (A,B) Representative IHC staining results of AHNAK2 in breast tumor ( n=40) and adjacent nontumorous tissues ( n=38). (C,D) Representative IHC staining results of AHNAK2 in colorectal tumor ( n=90) and adjacent nontumorous tissues ( n=90). (E,F) Representative images of the expression of AHNAK2 in esophageal glandular epithelium ( n=12) and tumor ( n=12). (G,H) Representative images of the expression of AHNAK2 in esophageal squamous epithelium ( n=9) and tumor ( n=9).

To address whether the expression pattern of AHNAK2 exists only in cervical adenocarcinoma and normal cervical gland, we further analyzed the expression of AHNAK2 in various types of adenocarcinomas and their corresponding normal glands from the HPA database, including breast, colon, lung, and pancreas. It was found that the expression of AHNAK2 was significantly higher in these adenocarcinomas than in normal gland tissues ( Supplementary Figure S3A–D). We also examined the expression pattern of AHNAK2 using breast cancer, colorectal cancer, and esophageal adenocarcinomas tissue microarrays. Consistent with the HPA database, the expression of AHNAK2 was significantly higher in adenocarcinomas than in normal gland tissues (breast cancer: *n*=40, *P*<0.01; Figure 3A,B and Supplementary Figure S3E; colon cancer: *n*=90, *P*<0.01; Figure 3C,D and Supplementary Figure S3F; and esophageal squamous: *n*=12, *P*<0.01; Figure 3E,F and Supplementary Figure S3G). Consistent with cervical squamous cell carcinoma, AHNAK2 is also highly expressed in both esophageal squamous cell carcinoma and normal squamous epithelial tissues ( *n*=9, *P*-value: NS; Figure 3G,H and Supplementary Figure S3G). Taken together, these data demonstrated that AHNAK2 is a potential biomarker for various adenocarcinomas.

### Knockdown of *AHNAK2* inhibits tumor progression and metastasis of adenocarcinomas

**Figure 4:**
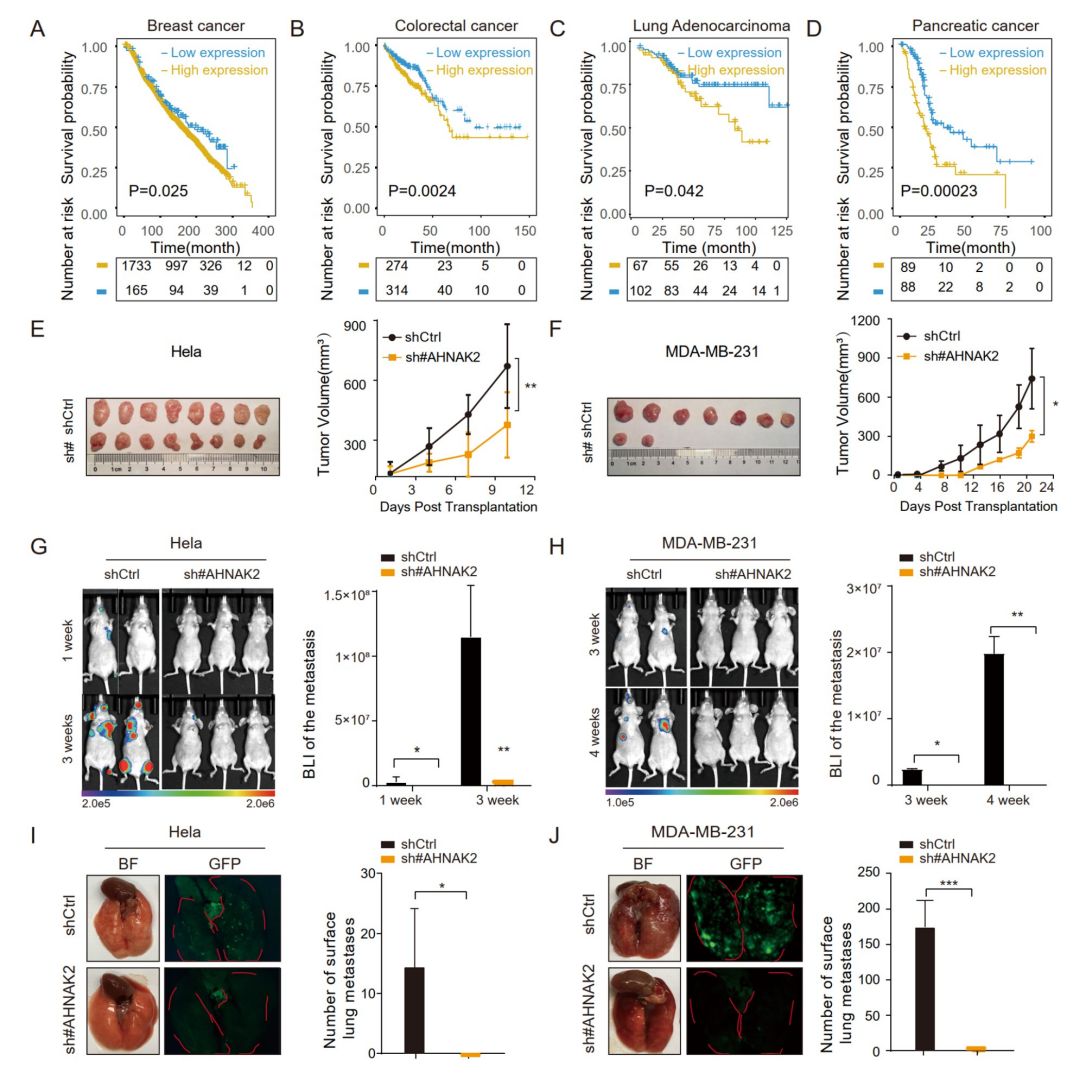
Figure 4 Knockdown of *AHNAK2* inhibits tumor progression and metastasis of adenocarcinomas (A–D) Kaplan-Meier analysis of the relationship between the expression of AHNAK2 and the overall survival prognosis of breast cancer patients (A), colorectal cancer patients (B), lung adenocarcinoma patients (C) and pancreatic cancer patients (D). (E,F) The tumor volume of each group was assessed post-transplantation. Images showed the dissected tumors after subcutaneous injection of Hela (E) and in situ injections of MDA-MB-231 (F). (G,H) Bioluminescence images of AHNAK2-deficient and the control nude mice collected after intracardial injection of Hela (G) and MDA-MB-231 (H) cells. (I,J) Representative lung metastasis areas and quantitative results of Hela cells (I) and MDA-MB-231 cells (J) after tail vein injection. * P<0.05, ** P<0.01, *** P<0.001.

We further tried to address whether AHNAK2 can be used as a biomarker only for adenocarcinomas and whether it functions in the tumor progression of adenocarcinomas. We first examined the clinical relevance of AHNAK2 expression and clinical patient prognosis by analyzing the database of the Cancer genome atlas (TCGA). Interestingly, patients with higher expression of *AHNAK2* were found to have poorer overall survival (OS) in breast cancer, colorectal cancer, lung adenocarcinoma, and pancreatic cancer ( Figure 4A–D). These results suggested that AHNAK2 may play a role in regulating the progression of breast cancer, colorectal cancer, lung adenocarcinoma, and pancreatic cancer.

To test this hypothesis, *AHNAK2* was knocked down in cervical adenocarcinoma cell line Hela and breast cancer cell line MDA-MB-231 using shRNA targeting *AHNAK2*. The knockdown efficiency was confirmed by RT-PCR ( Supplementary Figure S4A,B). *In vitro*, cell proliferation assay and 2D colony formation assay showed that *AHNAK2* knockdown inhibited cell growth of Hea ( Supplementary Figure S4C,E) and MDA-MB-231 ( Supplementary Figure S4D,F) cells. Sh#2-AHNAK2 was selected for subsequent xenograft assay as it was more effective. *In vivo*, two cell lines derived xenograft (CDX) models were used to study the role of AHNAK2 in adenocarcinoma tumor progression. One is to inoculate *AHNAK2*-knockdown MDA-MB-231 cells *in situ* into the mammary fat pads of nude mice, and the other is to inject *AHNAK2*-knockdown Hela cells subcutaneously into nude mice. *AHNAK2* knockdown dramatically delayed tumor formation and suppressed tumor growth in the Hela tumor xenograft model, as shown by reduced tumor volume and tumor size ( Figure 4E). Similar results were obtained from the MDA-MB-231 xenograft model ( Figure 4F).

AHNAK2 contains a PDZ (Postsynaptic density 95, PSD-85; Discs large, Dlg; Zonula occludens-1, ZO-1) domain, which is related to cytoskeletal dynamics of cell migration [23] . We therefore assessed the function of AHNAK2 on cell migration and invasion using wound healing and trans-well assays *in vitro* and evaluated the function of AHNAK2 on tumor metastasis using xenograft model *in vivo*. *In vitro*, wound-healing and trans-well assays showed that knockdown of *AHNAK2* significantly inhibited migration and invasion of Hela ( Supplementary Figure S4G,I) and MDA-MB-231 ( Supplementary Figure S4H,J) cells. *In vivo*, Luciferase-labeled *AHNAK2*-knockdown cells and control cells were injected into immunodeficient mice via the left ventricle. We observed that knockdown of *AHNAK2* significantly inhibited the metastatic burden of both Hela and MDA-MB-231 cells, as shown by bioluminescence imaging (BLI) ( Figure 4G,H). In addition, we observed consistent results in the lung metastasis established by injecting GFP-fluorescence labeled tumor cells into the tail vein. Significantly reduced lung metastatic burden was further illustrated by GFP-tagged tumor cells ( Figure 4I,J). Together, these results demonstrated that AHNAK2 is critical for the progression and metastasis of adenocarcinomas.

### AHNAK2 is involved in regulating DNA replication and up-regulating Bim expression

**Figure 5:**
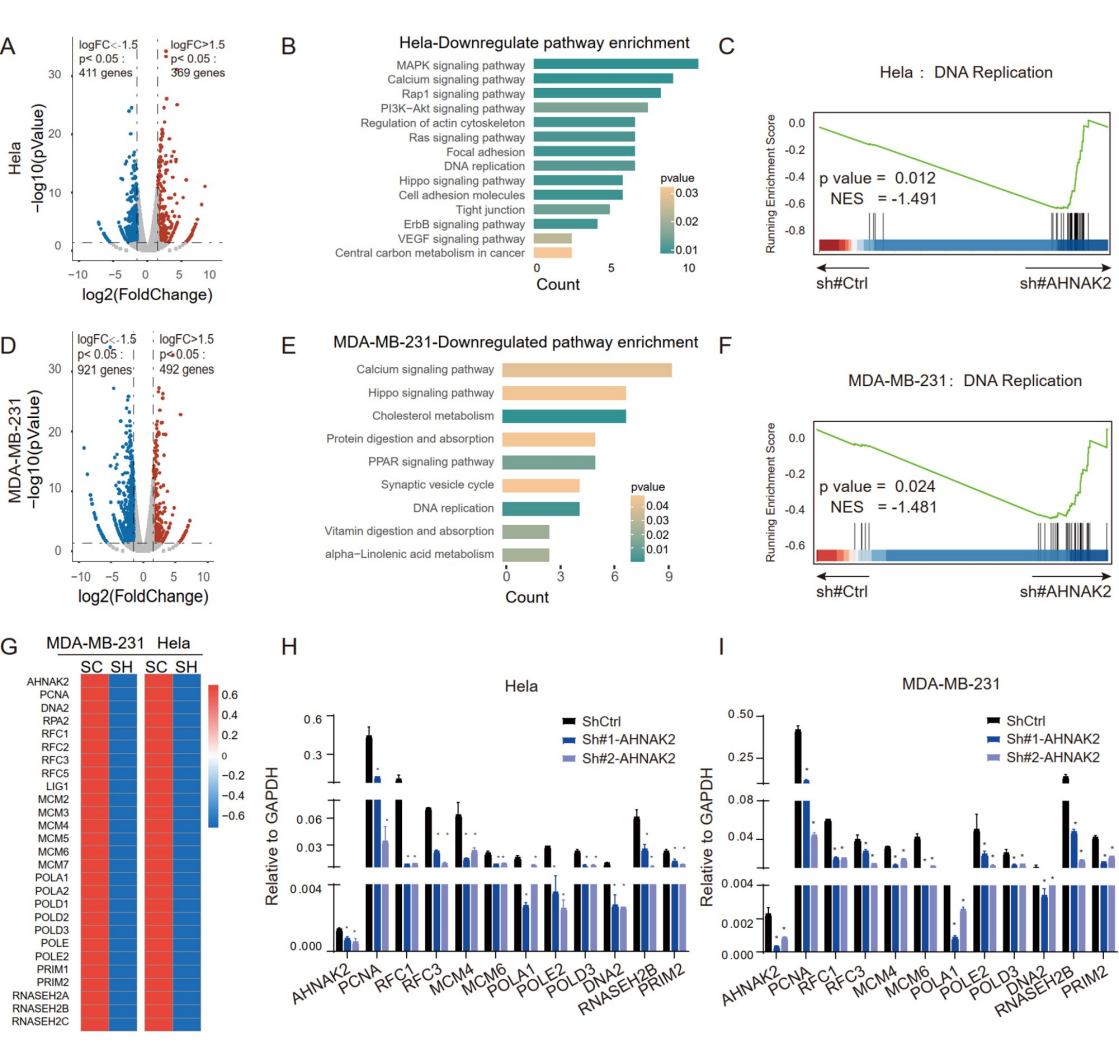
Figure 5 Analyses of signal pathway changes caused by knockdown of *AHNAK2* in Hela and MDA-MB-231 cells (A) Volcanic map of differential genes of AHNAK2-knockdown Hela cells. |Log FC|>1.5, P<0.05. (B) The histogram of KEGG pathway analysis of down-regulated genes of AHNAK2-knockdown Hela cells. (C) The same down-regulation pathway (DNA replication pathway) was enriched after AHNAK2 knockdown in Hela cells (NES=–1.491). (D) Volcanic map of differential genes of AHNAK2-knockdown MDA-MB-231 cells. |Log FC|>1.5, P<0.05. (E) The histogram of KEGG pathway analysis of down-regulated genes of AHNAK2-knockdown MDA-MB-231 cells. (F) DNA replication pathway was enriched after AHNAK2 knockdown in MDA-MB-231 cells (NES=–1,481). (G) Heat map showing the key genes of the DNA replication pathway after AHNAK2 knockdown in Hela cells and MDA-MB-231 cells. (H,I) RT-qPCR was used to validate the downregulated genes associated with DNA replication upon AHNAK2 knockdown in Hela (H) and MDA-MB-231 (I) cells. * P<0.05, ** P<0.01.

To understand the global biological effects of *AHNAK2* knockdown, we profiled the transcriptomes of *AHNAK2*-knockdown Hela and MDA-MB-231 cells by RNA-sequencing. Volcano plot analysis showed that *AHNAK2* knockdown induced the differential expression of 780 genes in Hela cell (|fold change|>1.5, *P*<0.05), of which 369 genes were up-regulated and 411 genes were down-regulated ( Figure 5A). KEGG pathway enrichment analysis showed that proliferation and migration signal pathways (such as MAPK, PI3K-AKT, DNA replication, Focal adhesion, and tight junction signals) were enriched in the down-regulated genes ( Figure 5B). For the MDA-MB-231 cells, volcano plot analysis showed that knockdown of *AHNAK2* caused expression changes of 1013 genes (|fold change|>1.5, *P*<0.05), of which 921 genes were significantly downregulated ( Figure 5D). KEGG and Gene Set Enrichment Analysis (GSEA) showed that gene signature of DNA replication signal was significantly downregulated in *AHNAK2*-knockdown MDA-MB-231 cells ( Figure 5C,E,F and Supplementary Table S2), which is consistent with the results in Hela cells. In addition, heat map analysis showed that the key genes related to DNA replication in Hela and MDA-MB-231 cells were significantly downregulated upon *AHNAK2* knockdown ( Figure 5G). RT-qPCR analysis further verified the results in Hela ( Figure 5H) and MDA-MB-231 ( Figure 5I) cells.

EdU is a thymidine nucleoside analogue which can replace thymidine (T) to penetrate into replicated DNA molecules during cell proliferation, and can be used to detect DNA replication activity. EdU staining analysis showed that knockdown of *AHNAK2* significantly reduced the proportion of EdU positive cells in Hela and MDA-MB-231 cells ( Supplementary Figure S5), indicating that knockdown of *AHNAK2* inhibited DNA replication. We further analyzed the TCGA database to explore the underlying mechanism of AHNAK2 in regulating cell behavior. The results showed that AHNAK2 deficiency may lead to the loss of contact between extracellular matrix and adjacent cells ( Supplementary Figure S6A–C), thus inducing anoikis which is closely related to cell migration and tumor metastasis [24]. In addition, we found that *AHNAK2* knockdown induced apoptosis of Hela and MDA-MB-231 cells ( Supplementary Figure S6D). To confirm that *AHNAK2* knockdown induces the anoikis, we performed western blot analysis to examine the expression of Bim. The results showed that knockdown of *AHNAK2* resulted in increased expression of Bim, indicating the occurrence of anoikis ( Supplementary Figure S6E). Together, these results suggested that AHNAK2 regulates tumor progression by mediating DNA replication and anoikis.

## Discussion

In this study, we used WGCNA to investigate the differentially expressed genes in SCC and AC of cervical cancer, and screened a specific gene *AHNAK2* related to the development and progression of cervical adenocarcinoma. The expression of AHNAK2 in cervical adenocarcinoma was significantly higher than that in the normal cervical glandular epithelium, but there was no significant difference between cervical squamous cell carcinoma and normal squamous epithelium. Interestingly, it was further found that AHNAK2 was not expressed in a variety of normal glandular epithelium, but highly expressed in a variety of corresponding adenocarcinomas, such as the esophagus, breast, and colon. Therefore, AHNAK2 can be used as a biomarker for adenocarcinomas. In functional studies, *AHNAK2* knockdown significantly inhibited the progression and metastasis of breast cancer and cervical cancer. Bioinformatics analysis suggested that the knockdown of AHNAK2 would damage the function of DNA replication. Our study demonstrated that AHNAK2 is a novel biomarker of adenocarcinomas, and has the potential to become a target for the treatment of adenocarcinomas.

AHNAK2 is a giant polypeptide which was first identified in 2004. It has the same common location and function as AHNAK in the cardiomyocytes of AHNAK null mice [23]. AHNAK2 belongs to the AHNAK family (or nucleoprotein AHNAK, which means giant in Hebrew). The AHNAK family includes AHNAK1 and AHNAK2 which are estimated to be about 700 kDa and 600 kDa respectively, and are all called giant proteins [ 25, 26] . In recent years, based on bioinformatics analysis, AHNAK2 has been reported to be involved in the carcinogenesis of a variety of cancers, especially adenocarcinomas. In pancreatic cancer, the meta-analysis or artificial neural network (ANN) analysis of transcriptome data revealed that AHNAK2 is a diagnostic and prognostic factor [ 27– 31] . In the analysis of genomic and epigenomic aberrations, AHNAK2 was revealed to be an oncogene of thyroid cancer, and the analysis of TCGA database further showed that AHNAK2 is a biomarker for the diagnosis and prognosis of thyroid cancer [ 32, 33] . Through the analysis of the public database, the oncogenic role of AHNAK2 in lung adenocarcinoma was also found [ 34– 36] . In addition, through the data mining analysis of multiple databases and the verification of key genes in clinical samples ( *n*=355) and cell lines, AHNAK2 was identified as a prognostic marker and oncoprotein of clear cell renal cell carcinoma [37]. No histological results were available in the reports on the role of AHNAK2 in squamous cell carcinoma, including uveal melanoma and esophageal squamous cell carcinoma [ 38, 39] .

Our study revealed that AHNAK2 is highly expressed in a large number of adenocarcinoma tissues, including cervical adenocarcinoma, breast cancer and colorectal cancer, compared with their normal tissue glandular epithelium. Together with the results reported in literature, our findings on the different expressions of AHNAK2 in various histological types (including squamous cell carcinoma and adenocarcinoma) suggest that AHNAK2 may be a unique oncogene and a suitable diagnostic marker in adenocarcinoma. In terms of functional research, the analysis of *AHNAK*-knockout mice showed that AHNAK1 and AHNAK2 compensate each other in physiological functions [23]. However, the study on the dominant structure and specific function of AHNAK2 is still limited. The two giant proteins of the AHNAK family share similar ternary protein structure. The short N-terminal fragment is connected to the repeat fragment, and the repeat fragment is organized into β-propeller protein and C-terminal. Although the N-terminal and C-terminal sequences of AHNAK are different, the N-terminal domain of AHNAK contains a PDZ domain related to protein-protein interaction [ 23, 40, 41] . In cells of nonepithelial origin, the subcellular localization of AHNAK is mainly limited to the nucleus and Golgi apparatus, while the localization of AHNAK in the cytoplasm or on the plasma membrane of epithelial cells depends on the formation of intercellular contacts [ 42, 43] . The nuclear exclusion of AHNAK is mediated by the export signal of PKB/AK phosphorylation [42]. The immunohistochemical results of our study showed that the localization of AHNAK2 is mainly in the cytoplasm of adenocarcinoma cells. Therefore, whether the transportation mode of AHNAK2 depends on nuclear export signals needs to be further investigated.

Genome instability is a hallmark of cancer, and DNA replication is the most vulnerable cellular process leading to tumorigenesis [44]. DNA replication and repair are crucial biological processes that ensure the accurate replication of the genome and minimize the errors in the transmission of genetic information to daughter cells [45]. Our research provides a clue that AHNAK2 may determine the occurrence and progression of cervical adenocarcinoma and breast cancer by regulating DNA replication. However, the underlying molecular mechanism of AHNAK2 in regulating DNA replication remains to be determined.

Nevertheless, there are some limitations in the study. For example, the tumor model used in this study could not fully reflect the clinical characteristics, and more clinically relevant models, such as patient-derived xenograft (PDX) or patient-derived organoid (PDO) models should be rendered in future studies. In the mechanistic study, the clues obtained from bioinformatics analysis need to be verified by further experimental biology. In conclusion, our study demonstrated that AHNAK2 is a biomarker of adenocarcinoma, and it is urgent to further explore its function and the underlying mechanism in the future.

## Supplementary Data

Supplementary data is available at *Acta Biochimica et Biophysica Sinica* online.

## Supporting information

22074Supplementary_Figures

22074supplementary_TableS1

22074supplementary_TableS2
