# Supplementary material for: AHNAK2 is a biomarker and a potential therapeutic target of adenocarcinomas: AHNAK2 is a biomarker for adenocarcinomas
Source: Acta Biochim Biophys Sin (Shanghai). 2022 Aug 19;54(11):1708–19. doi: 10.3724/abbs.2022112 (PMC9828698; doi:10.3724/abbs.2022112)
Supplement: 22074Supplementary_Figures [file 22074Supplementary_Figures.pdf]

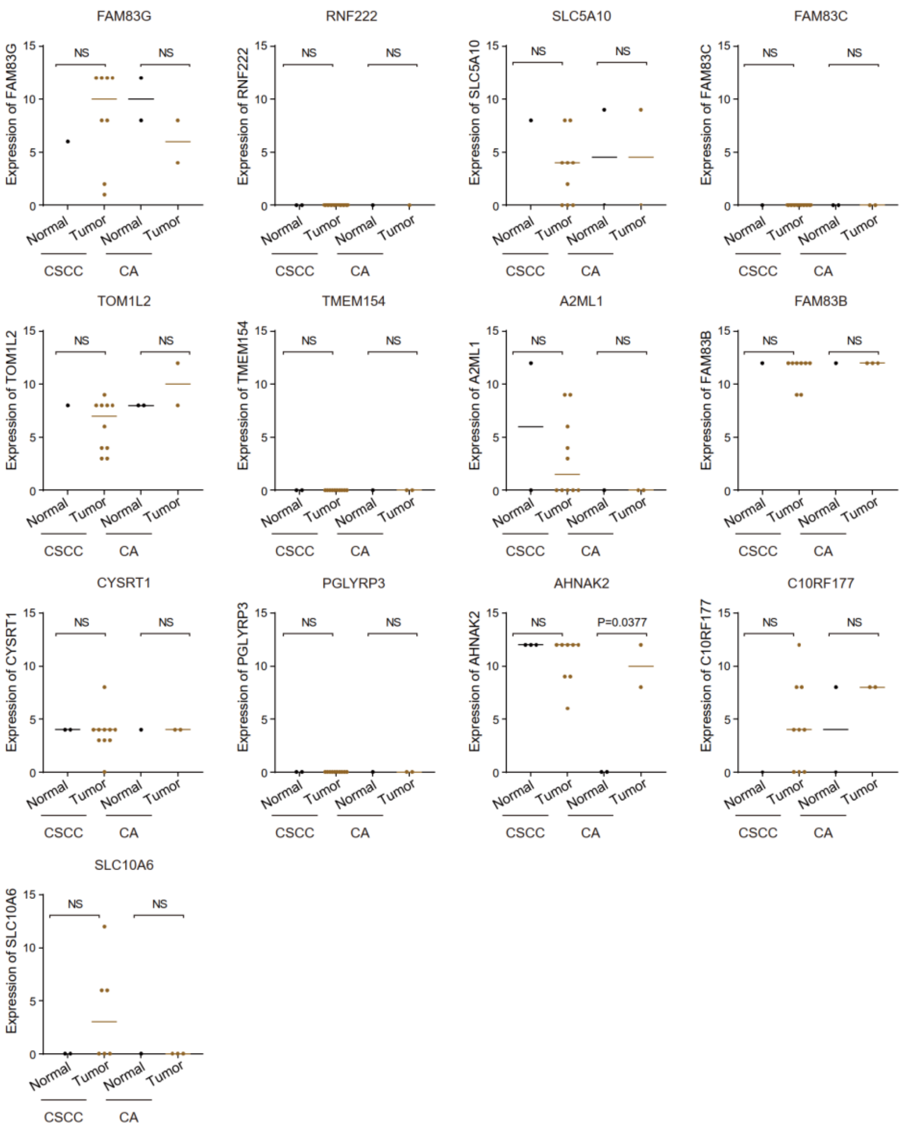

Supplementary Figure S1. The quantitative statistical results of FAM83G, RNF222, SLC5A10, FAM83C, TOM1L2, TMEM154, A2ML1, FAM83B, CYSRT1, PGLYRP3, RAET1E, AHNAK2, C1ORF177, and SLC10A6 on the HPA website CSCC, cervical squamous cancer. CA, cervical adenocarcinoma.

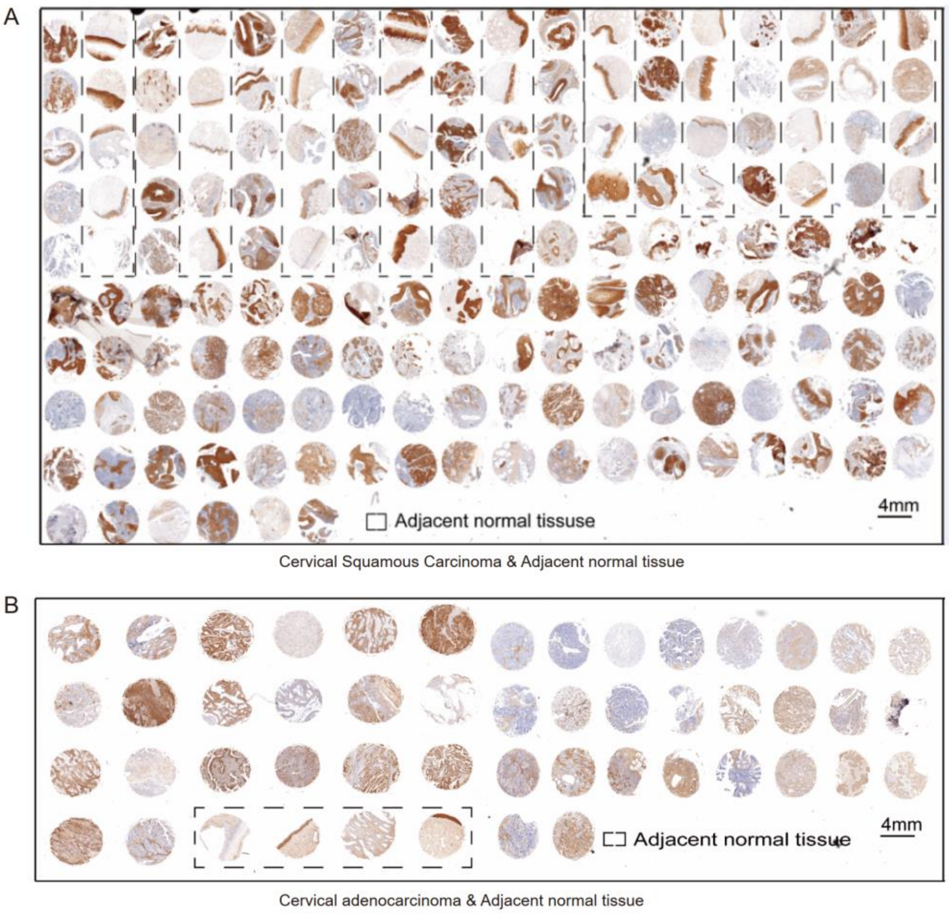

Supplementary Figure S2. AHNAK2 is highly expressed in cervical adenocarcinoma The expression of AHNAK2 in a tissue microarray of cervical squamous carcinoma and adjacent normal tissues (A), cervical adenocarcinoma, and adjacent normal tissues (B).

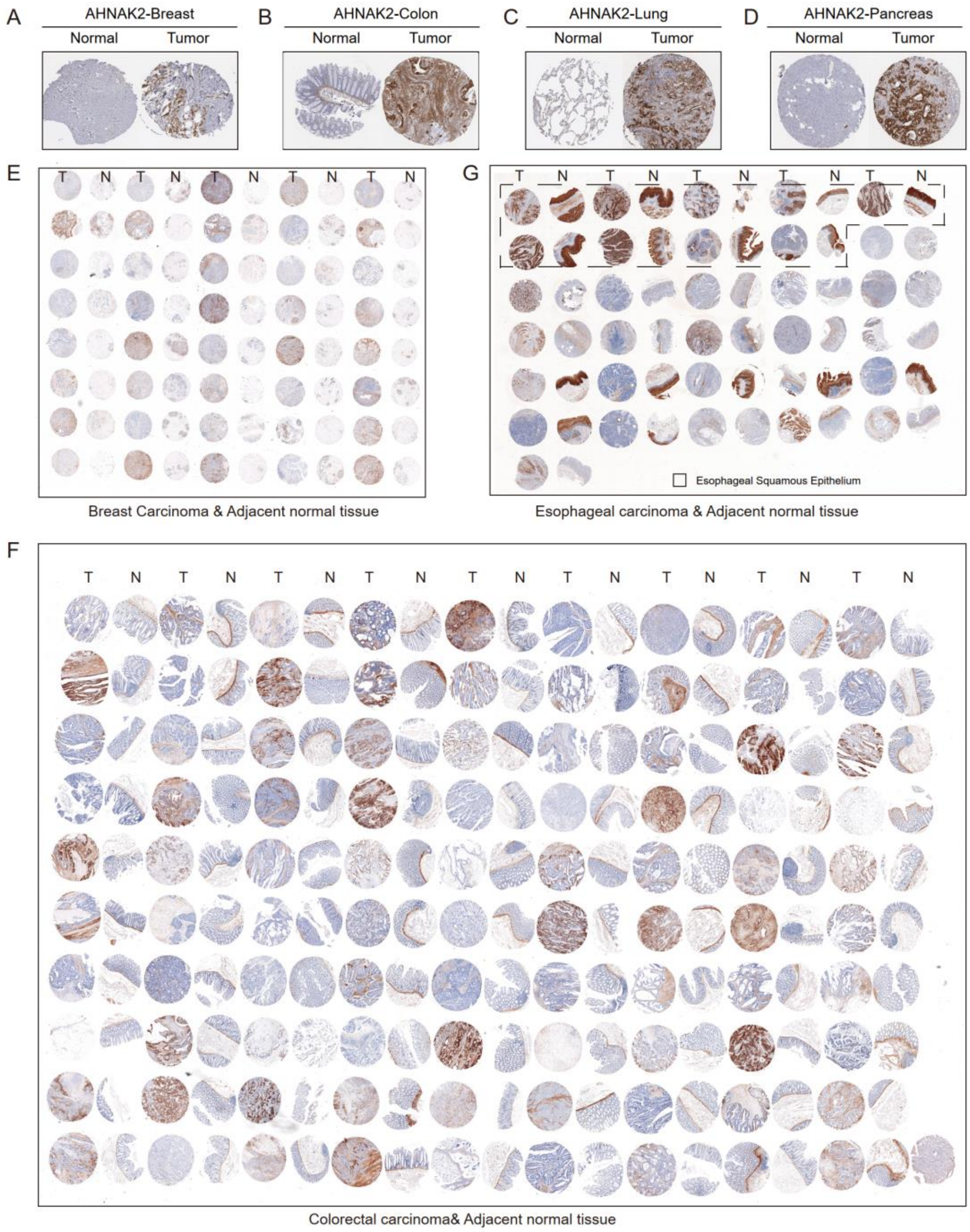

Supplementary Figure S3. AHNAK2 is highly expressed in various adenocarcinomas (A–D) The representative IHC results of AHNAK2 in breast cancer (A), colon cancer (B), lung adenocarcinoma (C), and pancreas (D), and their corresponding normal glandular epithelium tissues from Human Protein Atlas. (E,F) The expression of AHNAK2 in a tissue microarray of breast cancer (E), colorectal cancer (F), and esophageal carcinoma (G) and adjacent normal tissues detected by

IHC.

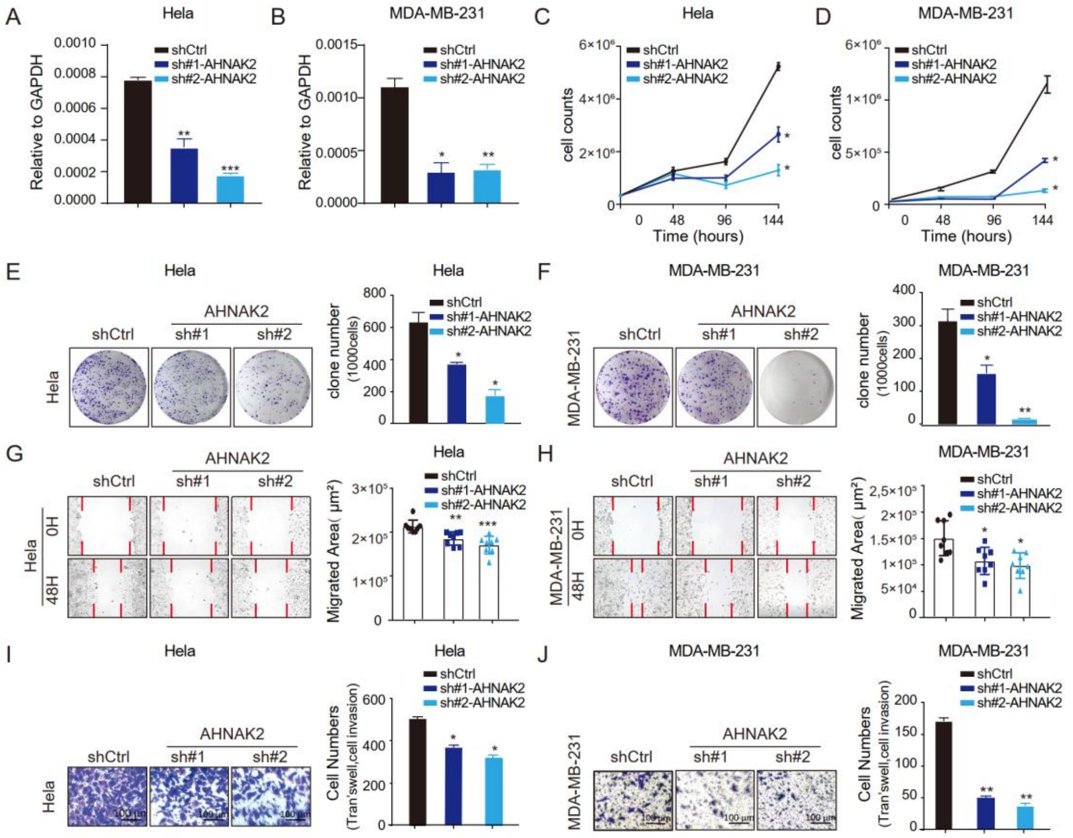

Supplementary Figure S4. Knockdown of *AHNAK2* inhibits proliferation and migration of adenocarcinoma cells (A,B) RT-qPCR analysis showed the knockdown efficiency of *AHNAK2* in HeLa cells (A) and MDA-MB-231 cells (B). (C,D) *AHNAK2* knockdown inhibited the proliferation of HeLa cells (C) and MDA-MB-231 cells (D). (E,F) Representative images of colonies formed by cervical adenocarcinoma cell HeLa (E) and breast cancer cell MDA-MB-231 (F) after 14 days culture. (G,H). Representative images of wound healing assay of HeLa cells (G) and MDA-MB-231 (H) after scratching. (I,J) Representative trans-well invasion assay of HeLa cells (I) and MDA-MB-231 cells (J) after *AHNAK2* knockdown. *P<0.05, **P<0.01, ***P<0.001.

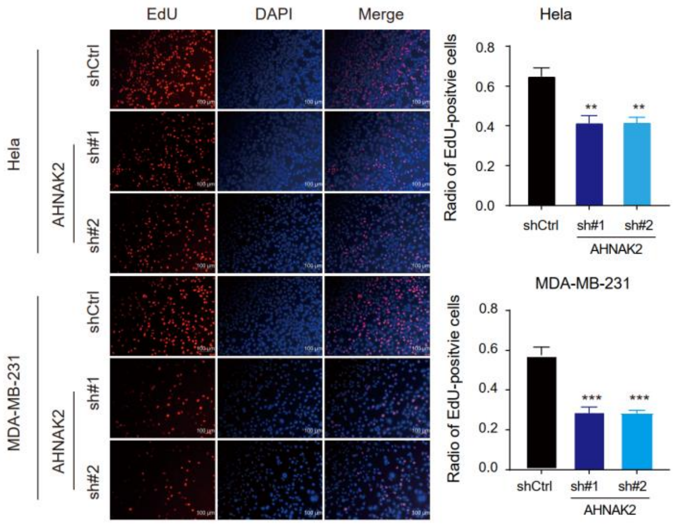

Supplementary Figure S5. Knockdown of *AHNAK2* inhibits the DNA replication activity in adenocarcinoma cells EdU staining assay showed that *AHNAK2* knockdown significantly reduced the proportion of EdU-positive cells in cervical adenocarcinoma cell HeLa and breast cancer cell MDA-MB-231. **P*<0.05, ***P*<0.01, ****P*<0.001.

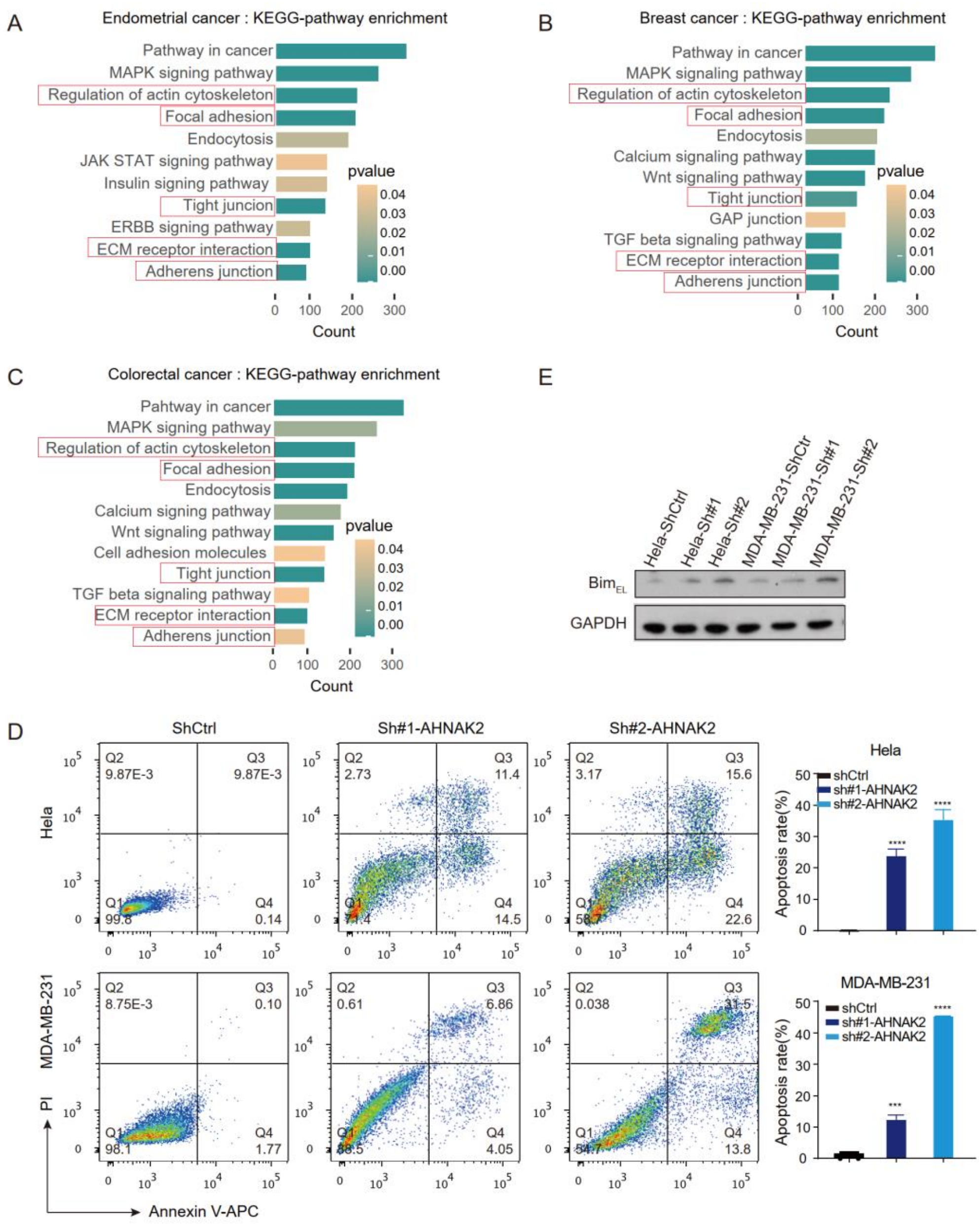

Supplementary Figure S6. *AHNAK2* knockdown induces the up-regulation of Bim expression (A). Histograms showing the KEGG pathways regulated by AHNAK2 in endometrial cancer. (B) The KEGG pathways regulated by AHNAK2 in breast cancer. (C) The KEGG pathways regulated by AHNAK2 in colorectal cancer. (D) Apoptosis of cervical adenocarcinoma cell Hela and breast cancer cell

MDA-MB-231 was induced by *AHNAK2* knockdown. (E) Western blot analysis was utilized to measure the expression of anoikis-related protein Bim after *AHNAK2* knockdown in cervical adenocarcinoma cell Hela and breast cancer cell MDA-MB-231. *$P<0.05$, **$P<0.01$, ***$P<0.001$.
