# Supplementary material for: AHNAK2 is a biomarker and a potential therapeutic target of adenocarcinomas: AHNAK2 is a biomarker for adenocarcinomas
Source: Acta Biochim Biophys Sin (Shanghai). 2022 Aug 19;54(11):1708–19. doi: 10.3724/abbs.2022112 (PMC9828698; doi:10.3724/abbs.2022112)
Supplement: 22074supplementary_TableS1 [file 22074supplementary_TableS1.pdf]

Supplementary Table S1.The genes and expressions of cervical cancer of TCGA database

gene	cor	gene	cor	gene	cor	gene	cor	gene
SDR9C7	0.75577	CYP4F22	0.60084	ADGRF4	0.53942	PLA2G4F	0.48519	SPAG17
FAM83G	0.73597	CAMSAP1	0.59433	GCOM1	0.53774	KRT80	0.48322	HRNR
CERS3	0.72082	BTBD11	0.59335	KRTDAP	0.53763	LGALS7B	0.48265	FLG2
RNF222	0.71143	THEM5	0.58687	BPIFC	0.53184	CCDC85C	0.47352	FAM46B
SLC5A10	0.71098	TMPRSS1	0.5833	TMEM184	0.53026	LYPD5	0.47261	MAL2
FAM83C	0.6794	GSDMA	0.57566	EHBP1L1	0.52266	ARHGEF3	0.47195	DHRS1
DENND2C	0.67939	FRMD6	0.57314	WDFY2	0.51568	LUZP6	0.47089	ANKRD35
TOM1L2	0.67572	NIPAL4	0.57247	WFDC12	0.51351	MAB21L3	0.46769	SPRR4
TMEM154	0.66624	KPRP	0.57181	IFFO2	0.51145	TM4SF19	0.46446	TINCR
A2ML1	0.66237	BNIPL	0.56907	LIPN	0.50988	TMPRSS1	0.45976	SOWAHB
FAM83B	0.65661	LCE3E	0.56598	SH3RF2	0.50742	MBOAT2	0.45971	SLC25A43
CYSRT1	0.64499	ARAP2	0.56361	MAST4	0.50337	OTOP3	0.45865	RAET1L
PGLYRP3	0.64297	SDR16C5	0.56351	FAM25BP	0.50276	TMPRSS1	0.45679	KCTD11
RDH12	0.63064	NIPAL1	0.55613	WFDC5	0.5005	CCDC64B	0.45381	LCE3A
RAET1E	0.6304	ACER1	0.55137	LINC0158	0.49897	S100A7A	0.45122	C12ORF7
**AHNAK2**	**0.61457**	DEFB103A	0.5481	C10ORF9	0.49867	PROM2	0.45037	HEPHL1
C1ORF177	0.61388	KRT78	0.54698	LIPK	0.49849	USH1G	0.45021	LCE6A
LINC00696	0.61306	LANCL3	0.54498	C15ORF6	0.49316	TPRG1	0.44936	DYNAP
PLA2G4E	0.60298	PHLDB3	0.5448	TCAF2	0.48565	AKR1B15	0.44856	LCE2C
SLC10A6	0.60165	CPEB2	0.53947	VSIG10L	0.48548	ADGRF2	0.44832	LCE2D

cor	gene	cor	gene	cor
0.44801	LINC0095	0.38576	ZNF276	0.3386
0.44756	TTC39B	0.3835	GJB4	0.33722
0.43845	DCUN1D3	0.37649	LINC0052	0.33712
0.42728	ASPG	0.37605	FABP12	0.32757
0.42329	CYP27C1	0.37482	MUC21	0.3199
0.41955	PNPLA1	0.37398	LINC0059	0.31704
0.41718	LCE1A	0.37173	CD164L2	0.30212
0.41318	TRIML1	0.36846	RPTN	0.30093
0.41297	GPR153	0.36777	RP1L1	0.29617
0.40844	OTOP2	0.36611	ATG9B	0.29206
0.40788	PLEKHA7	0.36415	CTXN3	0.28898
0.40586	ZBTB7C	0.35619	FAM92A1	-0.31813
0.40441	CLLU1	0.3548	GPX8	-0.40538
0.40017	FAM89A	0.35191	TMEM136	-0.41254
-0.48028	B3GNT8	0.35055	LINC0009	-0.42278
0.39658	CPNE8	0.34807		
0.39381	PRIMA1	0.34678		
0.3914	TMPRSS1	0.34601		
0.39115	RTKN2	0.34169		
0.38725	FAM102A	0.33875		
