# Supplementary material for: AHNAK2 is a biomarker and a potential therapeutic target of adenocarcinomas: AHNAK2 is a biomarker for adenocarcinomas
Source: Acta Biochim Biophys Sin (Shanghai). 2022 Aug 19;54(11):1708–19. doi: 10.3724/abbs.2022112 (PMC9828698; doi:10.3724/abbs.2022112)
Supplement: 22074supplementary_TableS2 [file 22074supplementary_TableS2.pdf]

Supplementary Table S2.The results of GSEA of Hela and MDA-MB-231

	ID	Description	setSize	enrichmen
hsa04740	hsa04740	Olfactory transduction	433	0.423775
hsa04630	hsa04630	JAK-STAT signaling pathway	162	0.498806
hsa00040	hsa00040	Pentose and glucuronate interconversions	33	-0.77768
hsa04380	hsa04380	Osteoclast differentiation	126	0.509432
hsa04975	hsa04975	Fat digestion and absorption	43	-0.7174
hsa04060	hsa04060	Cytokine-cytokine receptor interaction	292	0.422615
hsa03030	hsa03030	DNA replication	36	-0.73174
hsa04976	hsa04976	Bile secretion	89	-0.59884
hsa03440	hsa03440	Homologous recombination	37	-0.71148
hsa04621	hsa04621	NOD-like receptor signaling pathway	182	0.454697
hsa03320	hsa03320	PPAR signaling pathway	74	-0.59618
hsa04064	hsa04064	NF-kappa B signaling pathway	102	0.506864

NES	pvalue	p.adjust	qvalues	rank
1.257691	0.00734	0.504798	0.501497	3528
1.356205	0.007858	0.504798	0.501497	5299
-1.54635	0.009674	0.504798	0.501497	850
1.35819	0.009991	0.504798	0.501497	5837
-1.50125	0.011538	0.504798	0.501497	1138
1.213938	0.016509	0.55799	0.554341	3814
-1.48122	0.024731	0.696603	0.692047	7676
-1.38805	0.027437	0.713367	0.708702	2221
-1.45681	0.031181	0.752804	0.747881	8250
1.253585	0.033756	0.757712	0.752756	5639
-1.34744	0.03811	0.757712	0.752756	5075
1.324775	0.042573	0.799429	0.7942	4248
